# Malaria outbreak in Mbale: it´s the pits! a case study

**DOI:** 10.11604/pamj.supp.2022.41.1.31194

**Published:** 2022-03-23

**Authors:** Daniel Kadobera, Gloria Bahizi, Lilian Bulage, Benon Kwesiga, Stephen Ndugwa Kabwama, Alex Riolexus Ario, Julie Roberts Harris

**Affiliations:** 1 Uganda National Institute of Public Health, P.O Box 7272, Kampala, Uganda,; 2 Ministry of Health, Kampala, Uganda,; 3 College of Health Sciences, Makerere University School of Public Health, Kampala, Uganda,; 4 US Centers for Disease Control and Prevention, Kampala, Uganda,; 5 Division of Global Health Protection, Center for Global Health, US Centers for Disease Control and Prevention, Atlanta, United States of America

**Keywords:** Case study, malaria, *Plasmodium falciparum*, outbreak, Uganda

## Abstract

Malaria is a leading cause of morbidity and mortality in Uganda. In June 2019, the Uganda Ministry of Health through routine surveillance data analysis was notified of an increase in malaria cases in Bumbobi and Nyondo Sub-counties, Mbale District, which exceeded the action thresholds. We investigated to assess outbreak magnitude, identify transmission risk factors, and recommend evidence-based control measures. We defined a confirmed case as a positive malaria result using malaria Rapid Diagnostic Test or microscopy from 1 Jan 2019 to 30 Jun 2019 in a resident or visitor of Bumbobi or Nyondo Sub-county, Mbale District. We reviewed medical records to develop a line list for descriptive epidemiology. In a case-control study, we compared exposures between 150 case-persons and 150 age- and village-matched asymptomatic controls. We conducted environmental and entomological assessments on vector dynamics and behavior. We identified 7,891 case-persons (attack rate [AR]=26%). Females (AR=36%) were more affected than males (AR=25%). The 5-18 year age group (AR=26%) was most affected. The epidemic curve showed steady increase in malaria cases from March following intermittent rainfall from January, with short spells of no rainfall up to June. In the matched pair case-control analysis, 95% (143/150) of case-patients and 49% (73/150) of controls had soil erosion control pits near their homes that held stagnant water for several days following rainfall (AOR=18, 95%CI=7-50); Active breeding sites were found near and within homesteads with Anopheles gambiaeas the predominant vector. Increased vector breeding sites due to erosion control pits sustained by the intermittent rainfall caused this outbreak. We recommended draining of pits immediately after the rains and increasing coverage for bed-nets.

## How to use this case study

**General instructions:** case studies in applied epidemiology allow students to practice applying epidemiologic skills in the classroom to address real-world public health problems. The case studies are used as a vital component of an applied epidemiology curriculum, rather than as stand-alone tools. They are ideally suited to reinforce principles and skills already covered in a lecture or in background reading.

This case study has a facilitator guide and a participant guide. Each facilitator should review the Facilitator Guide, gain familiarity with the outbreak and investigation on which the case study is based, review the epidemiologic principles being taught, and think of examples in the facilitator´s own experience to further illustrate the points. Ideally, participants receive the case study one part at a time during the case study session. However, if the case study is distributed whole, participants should be asked not to look ahead.

During the case study session, one or two instructors facilitate the case study for 8 to 20 students in a classroom or conference room. The facilitator should hand out Part I and direct a participant to read one paragraph out loud, then progressing around the room and giving each participant a chance to read. Reading out loud and in turns has two advantages. First, all participants engage in the process and overcome any inhibitions by having her/his voice heard. Second, it keeps the all participants progressing through the case study at the same speed.

After a participant reads a question, the facilitator will direct participants to answer the question by perform calculations, construct graphs, or engage in a discussion of the answer. Sometimes, the facilitator can split the class to play different roles or take different sides in answering the question. As a result, participants learn from each other, not just from the facilitator. After the questions have been answered, the facilitator hands out the next part. At the end of the case study, the facilitator should direct a participant to once again read the objectives on page 1 to review and ensure that the objectives have been met.

**Prerequisites:** for this case study, participants should have received instruction or conducted readings in: Outbreak investigation; Intermediate epidemiology (interpreting odds ratio, epidemic curves, etc.); Basics of malaria epidemiology.

**Target audience:** trainees in the Uganda Field Epidemiology Training Program/Public Health Fellowship Program, other Field Epidemiology and Laboratory Training Programs (FELTPs), public health students, public health workers who may participate in rapid needs assessments and others who are interested in this topic.

**Level of case study:** intermediate or advanced

**Time required**: approximately 4 hours

**Language**: English

## Case study material

- Download the case study student guide (PDF - 559 KB)
- Request the case study facilitator guide
